# Social support, financial status, and depression among French healthcare workers one year after the onset of the COVID-19 pandemic

**DOI:** 10.1186/s12889-025-24066-4

**Published:** 2025-08-16

**Authors:** Charline Vincent, Pierre Chauvin, Honor Scarlett, Wissam El-Hage, Roberto Mediavilla, Cécile Vuillermoz

**Affiliations:** 1 Social Epidemiology, Mental Health and Addiction, ESSMA, Sorbonne University, INSERM UMR-S 1136, Pierre Louis Institute of Epidemiology and Public Health, 27, rue Chaligny, Paris, 75012 France; 2 UMR 1253, University of Tours, INSERM, IBrain, Tours, France; 3 Department of Psychiatry, Universidad Autónoma de Madrid (UAM), Madrid, Spain; 4 Instituto de Investigación del Hospital Universitario La Princesa - Hospital Universitario La Princesa, Madrid, Spain; 5 Centro de Investigación Biomédica en Red (CIBERSAM), Instituto de Salud Carlos III, Madrid, Spain; 6 UVSQ, CESP, Paris-Saclay University, Inserm, Paris, France

**Keywords:** Depressive symptoms, Healthcare professionals, COVID-19 pandemic, Mental health, Social support, Financial conditions, Social determinants

## Abstract

**Background:**

Early studies on factors associated with depressive symptoms among healthcare workers (HCWs) during the COVID-19 pandemic were primarily related to working conditions and exposure to the pandemic. Despite the socio-economic impact of the pandemic, research focusing on factors related to social support and financial situation was scarce.

**Objectives:**

This study investigated the association between HCWs’ financial and social support, and the presence of depressive symptoms in France one year after the pandemic’s onset.

**Methods:**

This study used data from a cross-sectional, online, voluntary survey conducted in France between April and October 2021 among a diverse, non-probability sample of HCWs. Data were calibrated according to the French Census of HCWs. Depressive symptoms (moderate to severe) were assessed using the 9-item Patient Health Questionnaire (score > 9). Four log-binomial regression models estimated prevalence ratios (PR) and their 95% confidence intervals (95%CI), taking into account working conditions, sociodemographics, and mental health comorbidities (post-traumatic stress disorder, anxiety, burnout).

**Results:**

Among the 655 respondents, 21% had moderate to severe depressive symptoms. Adjusted for working conditions and sociodemographics found significant associations between loneliness, lack of social support at work and worsening financial outcomes over the past year, with depression. After introducing mental health comorbidities, not only were they all associated with depression, but worsening financial outcomes and living alone remained associated with depression (PR = 1.23; 95%CI [1.04–1.45] and PR = 1.33; 95%CI [1.13–1.57], respectively).

**Conclusion:**

This study suggests that one year after the start of the pandemic, factors associated with depressive symptoms were related to social support and financial situation, unlike at the beginning of the pandemic when working conditions and exposure to COVID-19 were more studied and emphasized. These findings highlight the importance of considering the social determinants of health in studies and interventions aimed at improving the mental health of HCWs, and not just their working conditions.

**Supplementary Information:**

The online version contains supplementary material available at 10.1186/s12889-025-24066-4.

## Introduction

The COVID-19 pandemic has directly impacted health care services and their workers. In numerous countries, several systematic reviews have highlighted a high level of depression, anxiety and post-traumatic stress disorders (PTSD) among healthcare workers (HCWs) during the pandemic [1, 2]. One of the most prevalent mental health disorders among HCWs was depressive symptoms [2]. In 2024, a meta-analysis of 161 studies conducted in 49 countries (including 23.0% in Europe) estimated the overall prevalence of depressive symptoms among HCWs at 34% [3].

Studies on depression in HCWs have primarily focused on working conditions and exposure to COVID-19. They found an association between depression and working conditions such as being a nurse rather than a doctor or another professional [4], increased working hours [5], working in intensive care or an emergency medical unit [4], having a short-term position [6], and/or being a student [7]. They also found an association between depression and exposure to COVID-19 such as subjective exposure to the pandemic [8], having been tested positive for COVID-19 [9, 10] and having had relatives hospitalized for COVID-19 [9, 11].

Some studies have also focused on the relationship between depressive symptoms among healthcare workers and their sociodemographic characteristics and health comorbidities. On the one hand, being female [4, 11], young [8, 10, 11], having a low level of education, having young children [12] and, on the other hand, suffering from anxiety, post-traumatic stress disorder, burnout or history of chronic diseases [4, 11] and taking psychoactive medications [13] were associated with depressive symptoms in healthcare workers.

With the prolonged duration of the pandemic and its repeated waves, healthcare workers may have suffered from chronic stress, with subsequent consequences for their mental health status, either in terms of late-onset depression or in terms of longer-term chronic depression, one year (or more) after 2020. The meta-analysis led by Ciuluvica et al. in 2021 observed an increase of the prevalence of depression in HCWs [8]. However, most studies were conducted during the first wave of COVID-19 pandemic.

Only a few studies among healthcare workers (and none in France) have investigated associations between their depressive symptoms and their social support and their economic situation [5, 6, 10, 14, 15] although such associations have been well established in the general population [16, 17] - particularly after the COVID-19 pandemic [18–20] - and the great social diversity of HCWs makes such studies relevant. Among healthcare workers, studies conducted in 2021 focused either on financial resources [6, 10] or on social ties [12] or social support at work [14]. Their results remained limited due to the absence of multivariate analysis [10, 15] or the consideration of factors linked to the pandemic [6].

As it persisted, the COVID-19 pandemic led to a socio-economic crisis in which young people and women were the most affected worldwide [21]. In a U.S. study published in 2024, increasing economic insecurity was also reported among HCWs: those with a lower level of education or training (e.g., nursing assistants and home care workers) being at higher risk of financial loss [22]. Some HCWs, and in particular the most vulnerable, may have also experienced social isolation or loneliness [23], which are both risk factors for mental health and circumstances that can reduce their access to psychological support. In the face of a serious health crisis such as the Covid19 pandemic, it is therefore important to better understand the effect on HCWs mental health of both their working conditions and their social situation, since both are likely to be degraded.

The aim of this study was to investigate whether social support and financial resources were associated, or not, with depression in HCWs one year after the onset of the COVID-19 pandemic, when taking into account working conditions and exposure to the COVID-19.

## Materials and methods

### Study design and participants

This study used data collected by the French PSYCOVER survey (*PSYchological impact of the COVID-19 pandemic in healthcare workERs*) [24]. This cross-sectional survey of volunteers was carried out using an online self-administered questionnaire between April and October 2021, i.e. 13-months after the first surge of COVID-19 cases in France and coinciding with the fourth peak of the pandemic [25]. During May and June 2021, a curfew was enforced, and numerous establishments open to the public remained closed. Subsequently, in July 2021, a “health pass”, requiring proof of a negative PCR test within the last 72 h or full vaccination, was put in place to allow access to many public venues.

Inclusion criteria in PSYCOVER survey were being an adult who had work in a healthcare facility that had received patients with COVID-19 since the beginning of the pandemic, speaking one of the study languages (English and French), and having given an informed consent to participate. Participants were excluded if they were under 18 years of age, were not healthcare professionals, or if their facility had not received any patients since the beginning of the COVID-19 health crisis. Students and trainees were also eligible to participate. All types of HCWs could participate, i.e.– according to the French Public Health Code - medical professionals (physicians, midwives, dentists), pharmacists, medical physicists, and medical assistants (nurses, assistant nurses, childcare assistants, paramedics, physiotherapists, occupational therapists etc.).

Participants were recruited through: [1] calls to participate in social media platforms such as Facebook, Twitter, and LinkedIn; [2] communication with scholarly societies and professional associations (200 were contacted), asking them to inform and refer their members to the study website; [3] emails to institutional and academic partners; and [4] information to the managers of the 32 French university hospitals. Our final study population consisted of 655 HCWs.

### Outcome

According to the Diagnostic and Statistical Manual of Mental Disorders, 5th edition (DSM-5) [26], major depressive disorder is clinically defined as the experience of five or more of the following symptoms during the same 2-week period: decreased interest or pleasure, depressed mood, sleep disturbances, fatigue, weight loss or gain, feelings of worthlessness or guilt, difficulty concentrating, slowed thinking and physical movement and recurrent thoughts of death. At least one of the symptoms should be either depressed mood, or loss of interest or pleasure.

In our study, probable depressive symptoms in the preceding two weeks were assessed using the 9-item Patient Health Questionnaire (PHQ-9) [27, 28], in which people are asked to rate the frequency of each depressive symptom on a scale between 0 (not at all), 1 (several days), 2 (more than half the days), to 3 (nearly every day). The total PHQ-9 score ranges from 0 (indicating the absence of depressive symptoms) to 27 (indicating the most severe depressive symptoms). A cut-off score of ≤ 5 indicates the absence of any depressive symptoms, while scores between 5 and 9 suggest mild depression, scores between 10 and 14 moderate depression, scores between 15 and 19 moderately severe depression, and scores above 20 severe depression [27]. Cronbach’s alpha was 0.86 (95%CI: 0.85–0.88) for the PHQ-9 score in our sample. Our study outcome was the presence of moderate to severe depressive symptoms, i.e. having a score ≥ 10 [28, 29].

### Independent variables

Participants’ financial resources were assessed based on self-reported monthly household income (dichotomized according to its distribution as > 4000 € (first quartile) or ≤ 4000 €) and perceived financial evolution since the beginning of the pandemic (deteriorated/not changed or improved).

Social support was assessed by 5 indicators: participants’ perceived loneliness since the beginning of the pandemic (dichotomized into feeling very lonely or rather lonely versus not very lonely or not at all); family status (living alone: yes/no); perceived social support in everyday life (*“Can you count on someone*,* whether members of your household*,* other family members*,* friends*,* neighbors*,* co-workers*,* or other members of your community*,* to help you in your daily life?”*: yes/no); perceived psychosocial support at work *(“Do you know of a resource person in your institution who can help you deal with the psychosocial risks associated with your professional activities (excessive stress*,* psychological trauma*,* burnout*,* etc.)?”*: yes/no); and feeling stigmatized for being a healthcare professional by friends, family, neighbors and/or community members since the start of the COVID-19 pandemic.

### Potential confounding factors

Socio-demographic characteristics included 4 variables: gender, age (under 40 years old vs. over 40), education level (below MD or PhD versus higher) and having at least one child under the age of three.

Exposure to COVID-19 was assessed through the perceived exposure to COVID-19 (rated on a scale from 0 to 10, with 10 indicating very high exposure, and using a median cut-off of 7); having been tested positive for COVID-19 (at least once at the time of the survey) and having had relatives hospitalized for COVID-19.

Working conditions included occupation (nurse, physician, other); declaring that working hours had been increased since the beginning of the pandemic; working night shifts (often or always/sometimes or never); having been forced to change position (role, department or specialty) since the beginning of the pandemic; place of practice (hospital versus ambulatory practice); working in intensive care or emergency unit (yes/no); type of employment contract (temporary versus fixed-term contract or tenure position); having held this position for less than a year (yes/no); and being a student or a trainee (yes/no).

Concerning mental health comorbidities, symptoms of post-traumatic stress disorder (PTSD) were measured using the Posttraumatic Stress Disorder Checklist for the DSM-5 (PCL-5), validated in French [30]. A score of ≥ 33 indicates the presence of PTSD symptoms, which has been calibrated against the gold-standard Clinician-Administered PTSD Scale for the DSM-5 [31]. Symptoms of anxiety were measured using the 7-item Generalized Anxiety Disorder Scale (GAD-7). A score of ≥ 10 indicates the presence of anxiety [32, 33]. Burnout was assessed using the Maslach Burnout Inventory test (MBI) [34] which has been validated in French [35]. We chose to dichotomize burnout into two categories (yes/no) based on the method proposed by Maslach who considers burnout to be characterized by high emotional exhaustion (score > 29) and low personal accomplishment (score ≤ 33), or high emotional exhaustion (score > 29) and high depersonalization (score > 11) [36]. Finally, any history of psychotropic treatment for more than six months was also questioned.

### Statistical analyses

Because the sampling of PSYCOVER survey was non-probabilistic, there was an increased risk of selection bias. To partially counteract this, we calibrated our data using the margin adjustment method developed by INSEE (the French National Institute of Statistics and Economic Studies) [37]. We chose to perform this calibration based on profession (physicians vs. other HCWs) and sector of activity (hospital versus ambulatory care practice) using data from the second and third quarters of the 2021 French Employment Survey [38] conducted by INSEE, using the Icarus package [39]. We chose to weight according to these two variables to be more representative of the French HCWs population: 68.8% of physicians in the survey compared to 18.5% in French HCWs in 2019, and 41.6% working in ambulatory practice in the survey versus 26% in French HCWs in 2019 [40]. We did not weight our study population according to education level because this variable was redundant with occupation, nor did we do it according to gender and age since the distribution of our study population closely reflected that of the general HCWs population by gender and age.

Variables had missing values - between 4.6% and 9% - resulting in a total of 22% of the study population with missing data. To handle these missing values and reduce simulation error, we imputed 20 data sets using the Multivariate Imputation by Chained Equations [41] and survey R packages [42], while also considering calibration weights.

The linearity of the discrete variables was assessed using Shapiro-Wilk tests [43] and, where necessary, were transformed into categorical variables.

Descriptive statistics of the total sample were first conducted on the raw dataset. The sample size and percentages comparing the proportions of the different modalities were reported for each variable. Univariate and multivariate analyses were then conducted on both the imputed and weighted datasets, which yielded results similar to those obtained with the raw data. Associations between independent variables and moderate to severe depressive symptoms were tested using log-binomial regression models [44]. Prevalence ratios (PR) and 95% confidence intervals (CI) were reported for each variable. After conducting the univariate analysis, all covariates with a p-value ≤ 0.20 (except for age and gender in M3 and M4) were included into successive models: an initial one (M1) with independent variables only (financial resources and social support), M2 further adjusted on working conditions; M3 further adjusted (systematically) on gender, age and education; and the last one (M4) further adjusted on mental health comorbidities. Statistical significance was defined as a two-tailed p-value of less than 0.05 for all inferential analyses. Variance Inflation Factors (VIFs) [45] were computed for all predictors. All statistical analyses were performed using R Studio version 4.3.1v [46].

## Results

### Descriptive results

A total of 655 HCW participated in the first wave of this study, of which 74.8% were women (Table 1). Of the participants, 48.0% were under 40 years old, 26.8% had children under the age of three and 34.5% had a level of education below an MD or PhD. The sample consisted of 11.2% nurses, 71.6% physicians and 17.2% in other occupations. Regarding mental health status, 21.1% of participants (95% CI [17.99; 24.59]) exhibited moderate to severe depressive symptoms. Additionally, 19.4% (95% CI [16.37;22.78]) had anxiety symptoms, 8.7% (95% CI [6.65; 11.32]) moderate or high PTSD symptoms, 18.2% burnout symptoms (95% CI [15.21; 21.54]) and 30.3% (95% CI [26.67; 34.11] had a past history of psychotropic treatment for more than six months. Regarding their financial resources, 13.2% of participants reported that they had deteriorated since the beginning of the pandemic. Regarding their social support, a significant proportion of participants reported the absence of psychosocial support at work (47.4%) and/or that they had been stigmatized for being a HCW since the beginning of the COVID-19 pandemic (44.8%). Also, although at a lower frequency, many of them lived alone (19.6%) - and even more of them felt alone during the pandemic (29.7%) -without any social support in daily life (17.0%).

**Table 1:** Description of the study population

		*n*	%
Sociodemographic			
Gender	Male	164	25.2
	Female	487	74.8
Age	Over 40 years old	335	52.0
	Under 40 years old	309	48.0
Educational level	≥ MD or PhD	430	65.5
	< MD or PhD	225	34.5
Has children aged under 3 years	No	297	73.2
	Yes	109	26.8
Financial resources			
Monthly household income	> 4000 euros	405	64.8
	≤ 4000 euros	220	35.2
Perceived financial evolution SBP^1^	Not changed or improved	566	86.8
	Deteriorated	86	13.2
Social support			
Perceived loneliness SBP^1^	No	448	70.3
	Yes	189	29.7
Living alone	No	520	80.4
	Yes	127	19.6
Perceived social support in one’s daily life	Yes	529	83.0
	No	108	17.0
Perceived psychosocial support at work	Yes	340	52.6
	No	307	47.4
Feeling stigmatized for being an HCW SBP^1^	No	343	55.2
	Yes	278	44.8
Exposure to COVID-19			
Perceived exposure to COVID-19	Low	205	31.8
	High	439	68.2
Has been tested positive for COVID-19	No	491	76.1
	Yes	154	23.9
Any relatives hospitalised for COVID-19	No	563	87.3
	Yes	82	12.7
Working conditions			
Occupation	Physician	469	71.6
	Nurse	73	11.2
	Other	113	17.2
Increased working hours SBP^1^	No	221	33.8
	Yes	432	66.2
Working night shifts	Never or sometimes	569	87.5
	Often or always	81	12.5
Forced change of position	No change	490	76.0
	At least once	155	24.0
Place of practice	Ambulatory care practice	274	42.1
	Hospital	377	57.9
Working in intensive care or emergency unit	No	541	82.6
	Yes	114	17.4
Type of employment contract	Fixed-term contract or tenure position	296	47.7
	Temporary	324	52.3
Held this position for less than a year	No	507	78.5
	Yes	139	21.5
Student or trainee	No	575	88.3
	Yes	76	11.7
Mental health			
Depressive symptoms	Any to mild^2^	486	78.9
	Moderate to severe^3^	130	21.1
Post-traumatic stress disorder	No^4^	555	91.3
	Yes^5^	53	8.7
Anxiety symptoms	No^6^	495	80.6
	Yes^7^	119	19.4
Burnout	No	491	81.8
	Yes^8^	109	18.2
History of psychotropic treatment^9^	No	424	69.7
	Yes	184	30.3

*PHQ-9* 9 items Health-Patient Questionnaire, *PCL-5* Posttraumatic Stress Disorder Checklist, *GAD-7* Generalized Anxiety Disorder Scale, *MBI* Maslach Burnout Inventory test, *EE* emotional exhaustion, *PA* personal accomplishment, *DP* depersonalisation, *MD* Doctor of Medicine

^1^Since the beginning of the pandemic

^2^PHQ-9 score < 10; ^3^PHQ-9 score ≥ 10

^4^PCL-5 score < 33; ^5^PCL-5 score ≥ 33

^6^GAD-7 score < 10; ^7^GAD-7 score ≥ 10

^8^MBI score: EE > 29 and PA ≤ 33 or EE > 29 and DP > 11

^9^for more than 6 months

### Univariate analysis

In univariate analysis (Table 2), all the characteristics regarding participants’ pejorative financial resources and social support were significantly associated with moderate to severe depressive symptoms: lower household income (PR 1.56, 95% CI [1.12; 2.15]) and a reported deterioration of one’s financial situation (PR 1.93, 95% CI [1.35;2.77]), perceived loneliness (PR 3.01, 95% CI [2.17;4.16]), living alone (PR 1.61, 95% CI [1.15;2.28]), lack of social support in daily life (PR 2.24, 95% CI [1.62;3.09]), lack of psychosocial support at work (PR 1.67, 95% CI [1.19;2.35]) and having felt stigmatized as a HCW (PR 1.46, 95% CI [1.05;2.00]), respectively.

**Table 2:** Factors associated with moderate to severe depressive symptoms (univariate analysis)

		%	PR [95% CI]	*p*-value
Sociodemographic				
Gender	Male	18.8	ref	0.344
	Female	22.4	1.19 [0.82;1.72]	
Age	Over 40 years old	20.8	ref	0.709
	Under 40 years old	22.0	1.06 [0.78;1.43]	
Educational level	≥ MD or PhD	17.5	ref	0.001
	< MD or PhD	28.3	1.62 [1.21;2.17]	
Has children aged under 3 years	No	18.0	ref	0.986
	Yes	17.9	1.00 [0.62;1.60]	
Financial resources				
Monthly household income	> 4000 euros	18.6	ref	0.016
	≤ 4000 euros	28.9	1.56 [1.12;2.15]	
Perceived financial evolution SBP^1^	Not changed or improved	20.3	ref	< 0.001
	Deteriorated	39.3	1.93 [1.35;2.77]	
Social support				
Perceived loneliness SBP^1^	No	14.0	ref	< 0.001
	Yes	42.0	3.01 [2.17;4.16]	
Living alone	No	20.2	ref	0.007
	Yes	32.7	1.61 [1.15;2.28]	
Perceived social support in one’s daily life	Yes	17.38	ref	< 0.001
	No	39.8	2.24 [1.62;3.09]	
Perceived psychosocial support at work	Yes	17.6	ref	0.002
	No	29.3	1.67 [1.19;2.35]	
Feeling stigmatized for being an HCW SBP^1^	No	18.8	ref	0.025
	Yes	27.5	1.46 [1.05;2.00]	
Exposure to COVID-19				
Perceived exposure to COVID-19	Low	19.0	ref	0.207
	High	24.7	1.30 [0.89;1.90]	
Has been tested positive for COVID-19	No	22.0	ref	0.443
	Yes	25.5	1.16 [0.80;1.67]	
Any relatives hospitalised for COVID-19	No	22.0	ref	0.238
	Yes	28.8	1.31 [0.86;1.99]	
Working conditions				
Occupation	Physician	21.2	ref	0.404
	Nurse	23.9	1.26 [0.83;1.79]	
	Other	26.0	1.22 [0.83;1.83]	
Increased working hours SBP^1^	No	16.3	ref	0.017
	Yes	26.2	1.61 [1.10;2.37]	
Working night shifts	Never or sometimes	22.8	ref	0.856
	Often or always	23.1	1.01 [0.64;1.61]	
Forced change of position	No change	19.6	ref	< 0.001
	At least once	32.3	1.65 [1.19;2.29]	
Place of practice	Ambulatory care practice	19.8	ref	0.201
	Hospital	24.4	1.23 [0.87;1.76]	
Working in intensive care or emergency unit	No	21.9	ref	0.228
	Yes	27.3	1.25 [0.87;1.79]	
Type of employment contract	Fixed-term contract or tenure position	23.3	ref	0.791
	Temporary	22.3	0.96 [0.70;1.32]	
Held this position for less than a year	No	22.1	ref	0.401
	Yes	25.5	1.16 [0.81;1.65]	
Student or trainee	No	22.5	ref	0.477
	Yes	26.1	1.16 [0.76;1.77]	
Mental health				
Post-traumatic stress disorder	No^2^	15.7	ref	< 0.001
	Yes^3^	81.0	5.16 [4.11;6.48]	
Anxiety symptoms	No^4^	10.7	ref	< 0.001
	Yes^5^	64.8	6.08 [4.58;8.06]	
Burnout	No	13.1	ref	< 0.001
	Yes^6^	51.8	3.96 [2.97;5.28]	
History of psychotropic treatment^7^	No	16.2	ref	< 0.001
	Yes	34.9	2.15 [1.61;2.89]	

*PCL-5* Posttraumatic Stress Disorder Checklist, *GAD-7* Generalized Anxiety Disorder Scale, *MBI* Maslach Burnout Inventory test, *EE* emotional exhaustion, *PA* personal accomplishment, *DP* depersonalisation, *MD* Doctor of Medicine, *PR* prevalence ratio

^1^Since the beginning of the pandemic

^2^PCL-5 score < 33; ^3^PCL-5 score ≥ 33

^4^ GAD-7 score < 10; ^5^GAD-7 score ≥ 10

^6^MBI score: EE > 29 and PA ≤ 33 or EE > 29 and DP > 11

^7^for more than 6 months

Among potential confounders, an educational level below an MD or PhD was significantly associated with moderate to severe depressive symptoms, as was increased working hours, forced change of position and all four mental health comorbidities studied.

### Multivariate analysis

In the multivariate regression analysis (Table 3), participants’ income was not associated with moderate to severe depressive symptoms in any of the 4 adjusted models whereas a deteriorated financial evolution since the beginning of the pandemic was significantly associated, once our models being adjusted on sociodemographic. This association persisted even after taking into account mental health comorbidities also: aPR 1.23, *p* = 0.014, see M4).

**Table 3:** Multivariate associations between financial resources and social support and moderate to severe depressive symptoms: without and with step-by-step inclusion of confounding factors

	M1		M2		M3		M4	
	aPR [95% CI]	*p*-value	aPR [95% CI]	*p*-value	aPR [95% CI]	*p*-value	aPR [95% CI]	*p*-value
Financial resources								
Monthly household income ≤ 4000 euros	1.19 [0.80; 1.57]	0.517	1.15 [0.80;1.64]	0.451	0.88 [0.61;1.29]	0.528	0.87 [0.72;1.05]	0.161
Deteriorate financial evolution SBP^1^	1.39 [0.95; 2.02]	0.085	1.36 [0.92;2.00]	0.121	1.52 [1.06;2.20]	0.024	1.23 [1.04;1.45]	0.014
Social support								
Perceived loneliness SBP^1^	2.37 [1.61; 3.51]	< 0.001	2.21 [1.48;3.31]	< 0.001	2.19 [1.47;3.27]	< 0.001	1.25 [0.98;1.60]	0.075
Living alone	1.07 [0.73; 1.56]	0.736	1.06 [0.74;1.53]	0.740	1.08 [0.75;1.54]	0.680	1.33 [1.13.1.57]	< 0.001
Lack of social support in one’s daily life	1.24 [0.86; 1.78]	0.235	1.22 [0.85;1.75]	0.285	1.18 [0.84;1.65]	0.335	0.79 [0.61;1.01]	0.058
Lack of psychosocial support at work	1.38 [1.00;1.91]	0.050	1.38 [1.01;1.90]	0.040	1.40 [1.04;1.90]	0.028	1.14 [0.95;1.36]	0.153
Feeling stigmatized SBP^1^	1.11 [0.79; 1.54]	0.556	1.06 [0.74;1.51]	0.753	1.02 [0.72;1.49]	0.916	0.91 [0.76;1.08]	0.271
Working conditions								
Increased working hours SBP^1^			1.33 [0.92;1.95]	0.132	1.37 [0.95;1.98]	0.096	1.26 [0.99;1.61]	0.063
Forced change of position at least one			1.21 [0.86;1.69]	0.265	1.26 [0.91;1.74]	0.168	0.99 [0.87;1.13]	0.915
Sociodemographic								
Be a female					1.09 [0.78;1.51]	0.623	0.93 [0.85;1.03]	0.200
Age under 40 years old					1.14 [0.83;1.58]	0.421	1.14 [0.78;1.04]	0.900
Educational level < MD or PhD					1.41 [1.03;1.93]	0.034	1.33 [1.13;1.58]	0.001
Mental health comorbidities								
Post-traumatic stress disorders^2^							1.34 [1.05;1.70]	0.018
Anxiety symptoms^3^							3.49 [2.37;5.14]	< 0.001
Burnout^4^							1.53 [1.19;1.97]	< 0.001
History of psychotropic treatment^5^							1.32 [1.08;1.62]	0.007

Model 1 includes financial resources and social support variables. Model 2 further includes the 2 candidate variables of working conditions in univariate analysis. Model 3 was further adjusted on gender, age and educational level. Model 4 includes the same variables as Model 3, along with mental health comorbidities: post-traumatic stress disorders, anxiety symptoms, burnout and history of psychotropic treatment

*PCL-5* Posttraumatic Stress Disorder Checklist, *GAD-7* Generalized Anxiety Disorder Scale, *MBI* Maslach Burnout Inventory test, *EE* emotional exhaustion, *PA* personal accomplishment, *DP* depersonalisation, *MD* Doctor of Medicine, *aPR* adjusted prevalence ratio

^1^Since the beginning of the pandemic

^2^PCL-5 score ≥ 33

^3^GAD-7 score ≥ 10

^4^MBI score: EE > 29 and PA ≤ 33 or EE > 29 and DP > 11

^5^for more than 6 months

Regarding social support, perceived loneliness since the beginning of the pandemic was significantly associated with moderate to severe depressive symptoms in M1, M2 and M3. After adjusting for mental health comorbidities in Model 4, this association weakened and was no longer strictly significant (*p* = 0.075). In addition, significant associations of a lack of psychosocial support at work persisted in M1, M2 and M3, but no longer after adjustment for mental health comorbidities. Conversely, living alone appeared to be a factor significantly associated with depressive symptoms once mental health comorbidities were taken into account (aPR 1.33, *p* < 0.001, see M4). Variance Inflation Factors remained low (maximum VIF = 1.2), suggesting negligible collinearity between variables.

Age and gender were not associated with moderate to severe depressive symptoms, but this latter fact could be due to a significant association with educational level. Indeed, individuals with a level below an MD or PhD were at higher risk of depressive symptoms than MDs and PhDs (aPR 1.33, *p* = 0.001), and they are mainly nurses or nursing assistants, two largely feminized professions.

Interestingly, all four mental health comorbidities studied were associated with moderate to severe depressive symptoms in the fully adjusted model (M4): history of psychotropic treatment (aPR 1.32, *p* = 0.007), post-traumatic stress disorder (aPR 1.31, *p* = 0.018), burnout (aPR = 1.53, *p* < 0.001), and especially anxiety symptoms (aPR 3.49, *p* < 0.001).

## Discussion

Our study is the only one conducted in France and one of the very few worldwide, which analyzed the association between social support, financial resources, and depression in HCWs when taking into account their sociodemographic, working conditions and mental health comorbidities.

In 2021, in France, one year after the onset of the COVID-19 pandemic, 21.1% of the 655 HCWs included in the PSYCOVER survey exhibited moderate to severe depressive symptoms. It was estimated at 21.0% in a survey conducted in 2 Paris hospitals the same year [47].

Generally speaking, comparing prevalence estimates of depression among HCWs across different study is highly challenging. Luo et al. underline the extreme variability of these estimate in their meta-analysis [48]. Indeed, studies have been rarely comparable due to variations in study populations, assessment tools for depression and (possibly) other mental health disorders, and the consideration of peak epidemic working conditions. Furthermore, difference in local COVID-19 epidemiology and healthcare system saturation (or not) contribute to this heterogeneity.

However, our prevalence estimate is consistent with the 21% reported in Spain during the same period [49], but higher than those from other studies [50]. As mentioned above, this discrepancy may be explained by variations in the study populations. Many studies have been conducted among most exposed HCWs to the pandemic, such as those working mainly in emergency and intensive care units [6], in primary health centers [9], or in regions heavily affected by the pandemic [51]. Moreover, several studies have been conducted in a single medical center [5, 51]. The highest prevalence of depression seems to have been observed in particular contexts where healthcare facilities were completely overwhelmed by the first two epidemic waves (e.g., 41.7% in Jordan in 2021) [10].

Our estimated prevalence in French HCWs is much higher than the estimated 12.5% prevalence of depression in the general population in France during the same period [52]. This disparity underscores the particular vulnerability of healthcare professionals to mental health challenges, a concern previously highlighted by the WHO [53].

In the general population, it is well established that young people and/or people with low levels of education are more likely to suffer from depressive symptoms [8, 10, 11] and financial difficulties. Given the composition of our sample, which predominantly comprised physicians, a financially advantaged group (the threshold of 4,000 €/month we used is twice as high as the French median income), the absence of associations between income level and depression is not unexpected. Moreover, the majority of our sample were hospital-based HCWs (57.9%) - i.e. civil servants or those with long-term contracts, whose income is insured during sick leave. In contrast, the remaining 42.1% were independent, ambulatory caregivers for whom the financial distribution of French government aid remained unequal, with some health professionals not having benefited from it [54, 55]. Overall, rather than absolute level of income, our study found it was the perceived relative deterioration of income that was associated with depression in without being able to conclude on the direction of the association in such a cross-sectional study.

We found associations between some social support characteristics and depression, even after adjusting for socio-demographic factors and mental health comorbidities, which is in line with results of a longitudinal study conducted through the different COVID-19 pandemic waves in Singapore [5, 12]. Perceived loneliness and lack of psychosocial support at work were associated with depression symptoms in M1, M2 and M3. In M4, lack of psychosocial support at work was no longer significant, while living alone became significantly associated with depression and perceived loneliness approached significance. Multicollinearity does not seem to explain this attenuation (max VIF = 1.2). The observed changes in significance are therefore more likely due to shared variance and conceptual overlap among variables, rather than statistical collinearity per se (e.g. perceived loneliness and mental health comorbidities such as anxiety symptoms). These results demonstrate that feeling socially supported in in both personal and professional domains is associated with depression.

In the context of a health crisis such as the COVID-19 pandemic, our findings suggest that social support in personal and professional life is associated with depression among HCWs. Interpersonal relationships at work, including social support from management, play a crucial role. During the pandemic, the WHO recommended training health facility managers on psychosocial risks [53]. In occupational health in general, both employers and work organizations can contribute to improving mental health at work by creating an environment conducive to change [56]. It is unclear whether, in the medical and healthcare environment, practices address the challenges of HCWs’ mental health problems in acute and/or prolonged health crises such as COVID-19. One of the consequences of the COVID-19 pandemic in France was the creation in 2022 of a free and universal package for a few consultations with clinical psychologists, upon medical prescription (until then, consultations with psychologists were never covered by the French public health insurance system). Launched following the observation of the deterioration in the mental health of adolescents and young people during lockdowns, this package is accessible to the entire population but is not specifically dedicated to healthcare professionals. Additional measures should be implemented to strengthen empathy and support for healthcare professionals during this extreme health crisis.

Interestingly, while previous studies have found an association between HCWs’ exposure to COVID-19 [8–11] and working conditions [4–7] and depression, our study found limited evidence of these relationships. In univariate analysis, none of the 3 COVID-19 exposure measures were associated with depression, and only 2 of the 9 working conditions variables were associated with depression (increased working hours or any forced job change since the beginning of the pandemic). None of them were associated with depression in multivariate models. This may be explained by the fact that other studies did not include factors related to social support and financial status in their models. Conversely, Th’ng et al. [12] found no relationship between pandemic-related factors and depressive symptoms after adjustment for social support, which is in line with our results. Furthermore, the absence of significant associations between COVID-19 exposure, working conditions, and depressive symptoms may also reflect measurement limitations. Our exposure variables may not have fully captured the complex and multidimensional nature of healthcare workers’ exposure to the pandemic. Future studies could achieve greater precision by incorporating a more comprehensive assessment, including objective exposure (e.g., lack or inadequate access to personal protective equipment), subjective risk exposure (e.g., fear of contamination), geographical exposure (e.g., working in a high-incidence area). Moreover, employing validated scales such as the Fear of COVID-19 Scale would allow for more standardized and generalizable results.

Our study has some limitations. First, the PsyCOVer survey used a non-probabilistic sampling method, which may have introduced recruitment biases. The online data collection approach may have excluded individuals without computer access or with limited digital literacy. The survey was mainly disseminated through professional networks and institutional mailing lists, which may partly explain the overrepresentation of physicians in the sample (over 70%). To improve representativeness, all analyses were weighted using a calibration procedure based on the marginal adjustment method developed by INSEE, aligning the sample with the national distribution of healthcare professions in France. Second, the voluntary recruitment process may have introduced selection bias at initial participation. HCWs with more favorable working and social conditions, and especially those in good health and with lower exposure to stressors, may have felt less legitimate to participate due to the survey’s focus on the mental health of healthcare workers. Conversely, people who were sicker or more distressed may have been unable to participate [57]. Third, the presence of missing data should be considered. We attempted to minimize this by imputing the missing data. Finally, the cross-sectional design of the PSYCOVER survey impedes the establishment of causal relationships. The link between social isolation and depression, as well as that between financial situation and depression (e.g., depression leading to sick leave), may be bidirectional.

Our study also has several strengths. First, mental health problems were assessed using four reliable and standardized measurement scales, all validated within the French context. Second, the PSYCOVER survey was not limited to frontline hospital workers but also included community caregivers. It was also not limited to nurses and doctors, but addressed all health professions (although, in fact, the majority of respondents were physicians). Third, we used adequate methods to adjust this voluntary sample to the general population of HCWs and to impute missing data. Finally, log-binomial regression was preferred over logistic regression as it allows for a more straightforward interpretation of the estimated associations.

## Conclusion

Our study shows that, one year after the start of the COVID-19 pandemic, social support and financial difficulties were significantly associated with depressive symptoms among HCWs. After adjustment for other covariates, we did not detect a statistically significant independent association between pandemic-related work factors and depressive symptoms.

From a public health perspective, our study highlights the need to improve social and financial support for HCWs during major health crises. This involves securing HCWs’ financial resources, implementing preventive and management strategies psychosocial risks at workplace, and using community-based approaches to mitigate their loneliness and social isolation.

To enhance the mental health of HCWs, some studies suggest that emotional and psychological interventions can be effective, including: (a) psycho-education and training, such as booklets on how to recognize the signs of mental health disorders [58], workplace well-being promotion [59], or the organization of on-site trainings by psychologists [60]; (b) support and counselling by peers and/or multidisciplinary mental health teams operating in health care services [58, 59, 61], including group interviews; and (c) digital platforms, such as mobile applications for self-assessment of mental health status [62] and telephone helplines for listening and counselling [59, 62]. Additionally, several studies advocate for organizational changes, which may involve leadership and teamwork [58, 62], but also safer and healthier environments, including rest areas [59, 62, 63], and more accessible mental health resources, including psychological support [63]. However, our study introduces an important consideration regarding these interventions: while highlighting the importance of developing strategies for HCWs experiencing loneliness or financial hardship, it is known that socially disadvantaged individuals are less likely to participate in interventions than their more advantaged counterparts. Therefore, it is essential to avoid purely universal interventions without targeted actions to prevent exacerbating social health inequalities. Nevertheless, identifying these vulnerable populations, particularly in the workplace where these issues are often stigmatized, presents a significant challenge. Thus, a proportionally universal approach is warranted, combining universal actions for all HCWs with specific, tailored interventions for disadvantaged HCWs, who demonstrably experience poorer mental health.

## Supplementary Information

Supplementary Material 1.

## Data Availability

The data that support the findings of this study are available from the National Institute of Health and Medical Research (Inserm, “Institut national de la santé et de la recherche médicale”) but restrictions apply to the availability of these data, which were used under license for the current study, and so are not publicly available. Data is, however available from the authors upon reasonable request and with permission of the Inserm.
